# Strategic appeals and audience alignment in government short videos: A corpus-based analysis

**DOI:** 10.1371/journal.pone.0351639

**Published:** 2026-07-10

**Authors:** Hong Zhang

**Affiliations:** EIT Data Science and Communication College, Zhejiang Yuexiu University, Shaoxing, Zhejiang, China; NingboTech University, CHINA

## Abstract

This study examines how government short videos construct persuasive discourse through rhetorical strategies, using the Douyin account *Meili Zhejiang* as a case study. Drawing on audience-identification theory and Aristotle’s three rhetorical appeals (ethos, pathos, logos), it develops a text-centered rhetorical-computational framework that combines corpus-based methods and rhetorical analysis. The dataset covers 360 top-liked videos posted over one year and all publicly visible comments, with exact-text duplicates removed. Python-based lexical and sentiment analyses, together with concordance inspection in AntConc, are used to trace how persuasive appeals are realized in the textual dimension of the videos and how they align with audience orientations. Results show that pathos predominates (44.77%), expressed through affect-rich language of familial warmth, collective pride, encouragement, and urgency. Ethos (28.33%) is built through institutional roles and localized references, often strengthened by citizen voices, while logos (26.89%) appears in factual and quantifiable expressions that ground emotional and ethical claims. Sentiment in the associated comment corpus shows a generally positive tendency (56.7%), consistent with the audience profile and the rhetorical configuration identified in the corpus. The study offers a corpus-assisted rhetorical approach to analyzing persuasion in platform-mediated governance and highlights how communicative effectiveness is shaped by a calibrated construction of appeals aligned with audience dispositions in short-video communication.

## 1. Introduction

The rise of short video platforms has introduced a novel and dynamic channel for government communication, offering new opportunities for public information dissemination and citizen engagement. In this context, a central challenge for contemporary government communication is not only to convey messages with clarity and precision but also to capture public attention, foster interaction, and enhance the overall effectiveness of information delivery [1]. These platforms, characterized by brevity, rapid circulation, algorithmic curation, and interactive affordances, have enabled governments to engage in more direct and participatory communication, thereby reshaping institutional-citizen relationships in significant ways [2,3]. Against this broader platform context, the present study limits its analysis to the textual dimension of short-video discourse, specifically video texts and audience comments.

From a rhetorical perspective, government short videos are best understood as deliberate rhetorical acts. Rhetorical communication, by nature, is a strategic and audience-centered process that employs symbolic resources to influence cognition, attitudes, and behavior [4,5]. Government short videos exemplify this communicative mode: they are purposefully constructed, guided by persuasive intent, and designed to resonate with specific target audiences. Rather than simply transmitting information, these videos function as persuasive discourses aimed at mobilizing attention, fostering identification, and aligning public sentiment with institutional narratives. Importantly, in the interactive and user-driven ecosystems of short video platforms, rhetorical effectiveness is co-constructed through ongoing audience engagement, feedback, and relational negotiation between message sender and receiver [6]. This interactivity reinforces the fundamentally audience-oriented nature of government short videos, where persuasive success depends not only on message design but also on the capacity to align rhetorical strategies with user preferences, expectations, and participatory behaviors.

Existing scholarship on government short videos, largely situated within journalism, communication, and political science, has examined them through lenses such as political communication, public relations, digital storytelling, social-media engagement, crisis communication, and audience reception. While these studies have illuminated technological affordances, dissemination patterns, content typologies, and engagement metrics [7–10], the field remains predominantly qualitative and has yet to systematically employ corpus-based methods. Moreover, the rhetorical and persuasive mechanisms of short-video discourse remain insufficiently specified. Few have systematically examined how persuasive appeals are constructed in government short videos, how they operate through discursive strategies, and how they interact with diverse audiences [11,12].

Drawing on Aristotle’s classical triad of rhetorical appeals—ethos (credibility), pathos (emotional resonance), and logos (logical reasoning), this study examines government short videos as persuasive discourse. By employing these appeals as a heuristic framework, the analysis highlights the rhetorical construction of meaning and maps the persuasive resources embedded in digital governance communication. Meanwhile, the analysis also incorporates the concept of audience identification from modern rhetorical and communication theory, which argues that persuasion is more effective when audiences perceive shared values, experiences, or goals with the communicator [13,14]. In the context of government short videos, identification is effectively cultivated through a synergy of emotionally compelling narratives (pathos), credible representation of institutional actors (ethos), and transparent, logically structured messaging (logos). Together, this integrated framework bridges rhetorical theory and contemporary scholarship on digital political communication [15,16] and supports a more nuanced understanding of how persuasive strategies are aligned with audience dispositions in platform-mediated governance.

Within this theoretical framework, this study investigates the persuasive dimensions of government short videos through the case of *Meili Zhejiang*, the official Douyin account of Zhejiang province. It explores how governmental actors mobilize textual and discursive resources to construct persuasive narratives, and how these strategies are designed to resonate with audience dispositions and norms of interaction on short video platforms. Rather than focusing solely on content dissemination, the study emphasizes how rhetorical appeals are configured to foster identification and communicative alignment with the target audience. To this end, the study is guided by the following research questions:

RQ1: How are Aristotle’s rhetorical appeals—ethos, pathos, and logos—realized in the textual discourse of government short videos?

RQ2: How do these rhetorical strategies align with audience dispositions and contribute to the construction of identification in government short video communication?

Methodologically, the study combines corpus-assisted discourse methods with qualitative rhetorical interpretation to examine the linguistic realization of rhetorical appeals and audience response patterns in video texts and comments. The findings contribute to rhetorical and communication scholarship by applying classical persuasion theory to the context of digital governance, offering practical implications for enhancing the efficacy of government short video communication.

## 2. Literature review

### 2.1. A rhetorical perspective on government short video communication

Rhetoric, once rooted in the classical traditions of persuasion and public speaking, has since expanded into a multidisciplinary field that examines how symbolic resources are strategically used to shape meaning and influence public reasoning [17]. Its influence permeates contemporary communication and media studies, maintaining enduring relevance through both Aristotle’s rhetorical framework and a growing emphasis on audience-centered discourse [5]. From a rhetorical perspective, the dissemination of government short videos constitutes a rhetorical act shaped by sociopolitical and cultural contexts, designed to orient public perception and foster audience engagement.

This rhetorical nature manifests in several interrelated dimensions. First, government short videos are designed with clear communicative intentions, whether to persuade, inform, legitimize, or mobilize, and this underscores their purpose-driven rhetorical orientation [18]. Second, the content is carefully tailored to the audience’s values, expectations, and media habits, reflecting rhetoric’s enduring emphasis on audience adaptation [19]. Third, the interactive affordances of short video platforms, such as likes, comments, and shares, encourage participatory engagement, thereby facilitating audience identification, a key rhetorical mechanism for building alignment and trust [15,20]. Finally, their situational relevance, often aligned with trending topics or policy events, illustrates the rhetorical significance of kairos, or the opportune moment of communication.

Recent research has approached government short videos from diverse angles, including political communication [11,21,22], public relations [2,23], digital storytelling [24,25], audience interaction and social media engagement [12,26,27], media convergence [28–30], crisis communication [31], among others. Collectively, these studies underscore the strategic importance of short videos in modern governance and citizen interaction.

Methodologically, most studies rely on qualitative approaches such as discourse analysis [32], semiotics [33], and case studies [13], but this predominance also indicates a degree of methodological homogeneity. Although discourse analysis and semiotics are traditionally qualitative, they can be extended through corpus-based techniques to incorporate quantitative and computational dimensions. This imbalance suggests the value of more diverse methodological designs that combine interpretive depth with empirical breadth, an orientation adopted in the present study.

Moreover, the rhetorical and persuasive mechanisms underlying official video narratives are still insufficiently examined. Employing rhetorical theory [16] offers a valuable lens for analyzing how symbolic strategies construct institutional credibility, foster audience identification, and generate persuasive appeals. This perspective informs the analytical framework of the present research, which integrates rhetorical analysis with corpus-based computational methods to capture both qualitative depth and quantitative scope.

### 2.2. Audience in rhetorical theory and government short videos

In rhetorical theory, the audience is not merely a passive group but an active agent essential to persuasion. Perelman and Olbrechts-Tyteca [34] define the audience as those whom the rhetor seeks to persuade through discourse, while Bitzer [35] emphasizes that rhetorical audiences are capable of being influenced and of mediating change. Subsequent scholars reinforce this view, highlighting the audience’s capacity for action and transformation [4,36,37].

As rhetoric is fundamentally audience-oriented, persuasion requires the rhetor to align discourse with the audience’s values, beliefs, expectations, and communicative preferences. Burke’s [15] concept of “identification” underscores this alignment, arguing that effective persuasion arises when rhetors identify with their audience by adopting shared language, imagery, attitudes, and symbolic forms [38]. In the contemporary media environment, however, the audience is no longer a singular or clearly defined entity. With the rise of mass communication, rhetorical discourse increasingly addresses diverse and fragmented publics. Covino and Jolliffe [39] further observe that rhetors often construct an “imagined” audience by summoning shared interests, knowledge, and needs. This imagined construct guides rhetorical choices even when the actual audience is diffuse or unknown.

In the context of government short videos, audience-centered communication becomes essential. These videos aim to inform, engage, and build rapport with diverse publics. Effective message design must be sensitive to audience characteristics, values, and media habits [40,41]. Rhetorical strategies should be adapted to foster identification and engagement across social groups. To investigate how rhetorical strategies are tailored to audiences in government short videos, this study conducts an audience-oriented rhetorical analysis focusing on audience profiles, attitudes, and interaction patterns. This analysis clarifies how persuasive appeals are realized in the textual and linguistic components of government short videos and how audience alignment is reflected in observable engagement patterns and comment responses. By linking rhetorical strategies with audience identification, the study aims to show how particular rhetorical constructions are associated with public endorsement and communicative impact in official digital discourse.

### 2.3. Aristotle’s persuasive appeals in government short videos

Building on the audience-centered orientation, Aristotle’s rhetorical theory offers a systematic account of persuasive appeals. Aristotle [42] identified ethos, pathos, and logos as the three modes of persuasion, each addressing a distinct dimension of rhetorical effectiveness. Ethos appeals to the speaker’s projected good character, authority, and virtues, thereby establishing their credibility [43]. Pathos appeals to the emotions and feelings of the audience, evoking a specific emotional response that aligns with the rhetor’s message. Logos, the final mode of persuasion, relies on logical reasoning, facts, and evidence to appeal to the audience’s rational faculties and demonstrate the validity of the rhetor’s claims [43,44]. These three modes of persuasion are interdependent and often operate in combination to produce a more compelling rhetorical discourse.

Beyond the classical formulation, ethos has also been conceptualized and measured in the source-credibility tradition of communication research, where credibility judgments are typically treated as multidimensional (e.g., competence, trustworthiness, and goodwill) and consistently influence persuasive outcomes [45]. At the same time, established scholarship on social evaluation and political communication posits that credibility is often relational. It can be built not only through positive self-presentation but also by positioning one’s claims against alternatives in ways that subtly weaken competing actors or interpretations. D’Errico, Poggi, and Vincze [46], for instance, argue that discrediting commonly works by casting doubt on an opponent’s competence, benevolence, or dominance. D’Errico and Poggi [47] further note that such evaluative conflict is frequently conveyed indirectly through framing choices, implied contrasts, or other contextual cues rather than through overtly negative wording alone. In this study, ethos is examined mainly in its affirmative sense through high-frequency lexical patterns. Relational or discrediting cues are not emphasized because the account’s positive-energy orientation and the monthly most-liked sampling are likely to foreground audience-endorsed, harmonious discourse; furthermore, overt discrediting is incongruent with the communicative strategies of government entities addressing a broad public. This point is further revisited in the Discussion.

The three appeals are crucial in the context of government short videos. First, ethos is established through the credibility and reputation of the news source or government agency, which serves to enhance the audience’s trust in the information provided. Second, pathos often plays a particularly significant role in the context of short videos due to the need to quickly capture the audience’s attention and evoke an immediate emotional response. Such affective framing may evoke feelings such as warmth, urgency, collective pride, or concern, which can strengthen involvement and encourage interaction with the content. Third, government short videos employ logos through factual evidence and concise reasoning, helping audiences interpret the information presented and assess its validity and relevance.

Building on this framework, this study examines high-frequency lexical patterns in *Meili Zhejiang*’s government short videos to show how ethos, pathos, and logos are embedded in the videos’ textual components and how these appeals are associated with audience responses. This approach offers insights into the relationship between persuasive strategies, viewer engagement, and communicative goals.

## 3. Methodology

### 3.1. Research design

This study employs a rhetorical framework to investigate the communication strategies of government short videos, with the Douyin account *Meili Zhejiang* as the focal case. Two theoretical lenses guide the analysis: the rhetorical concept of audience identification and Aristotle’s three persuasive appeals. To anchor interpretation in empirical evidence, the study integrates corpus-linguistic methods, building parallel corpora of video texts and audience comments from *Meili Zhejiang*. Python-based computational tools are then applied to extract linguistic features and audience attitudes, thereby providing quantitative support for rhetorical interpretation. By combining corpus-based methods with rhetorical interpretation, this design provides empirical support for examining how persuasive strategies align with audience dispositions and contribute to communicative effects [48].

### 3.2. Data and sampling

The dataset is drawn from the official Douyin account *Meili Zhejiang* (literally “Beautiful Zhejiang”), which has become one of the most influential provincial-level government accounts in China. With over 14.5 million followers and a steady record of content production and user engagement, the account provides a representative case for examining rhetorical strategies and audience responses in government short video communication [49,50].

The sample comprises the 30 most popular videos per month measured by the number of likes (rather than views) between April 1, 2023, and March 31, 2024, yielding 360 videos in total. Likes were selected because they provide observable indicators of audience approval, whereas view counts primarily reflect exposure and may be inflated by platform distribution. This design allows the study to examine rhetorical patterns in videos with relatively high levels of visible audience approval. Given the platform’s visibility and ranking mechanisms, the findings should be interpreted as characterizing high-approval, high-visibility communication rather than the account’s full routine output.

The inclusion and exclusion criteria were applied consistently during corpus construction. For each selected video, the video-text corpus included all retrievable textual materials, including transcribed speech from the video audio, subtitles or on-screen text, and accompanying post descriptions when they contained substantive verbal content. Non-textual elements, URLs, hashtags, emojis, platform metadata, and purely decorative symbols were removed during preprocessing. Videos were excluded only when their textual component was unavailable or insufficient for lexical analysis after preprocessing.

For each sampled video, all publicly visible comments under the video were collected during data collection. To reduce noise and avoid over-counting repeated content, exact-text duplicate comments were removed prior to analysis. Separate corpora were then constructed for video texts and comments. The content corpus contains 24,432 words and 30,926 tokens, while the comments corpus contains 591,227 words and 1,081,857 tokens, based on AntConc corpus statistics. These materials provide a comparable textual basis for examining production discourse and audience response within the same communicative environment.

### 3.3. Analytical procedures

The analysis proceeded in three steps. First, audience profiles and engagement metrics (likes, comments, shares) were collected from the Feigua Data platform (https://www.feigua.cn) to outline user demographics and interaction intensity.

Second, the comment corpus was computationally processed in Python. Initial preprocessing involved text cleaning and Chinese word segmentation using the Jieba library, followed by stopword removal using the Harbin Institute of Technology (HIT) stopword list. These steps supported frequency-based summaries and the generation of word-cloud visualizations to highlight salient concerns. Subsequently, sentiment analysis was conducted using the SnowNLP library, an open-source Python library for simplified Chinese natural language processing, with a targeted domain-specific calibration step.

To align the sentiment module with the linguistic characteristics of the present corpus, a manually constructed seed set consisting of 90 comments (30 clearly positive, 30 neutral, and 30 negative) was used for calibration. These seed comments were selected only when their affective orientation was unambiguous; mixed, ironic, or highly context-dependent expressions were excluded. Based on this calibration, sentiment scores were classified into three categories using empirically refined thresholds: scores below 0.40 were coded as negative, scores above 0.60 as positive, and those between 0.40 and 0.60 as neutral. After preliminary spot checks, the calibrated model was applied to the full corpus.

Given that automated classification may encounter difficulties with implicit attitudes or irony, the results are interpreted as descriptive indicators of broad audience tendencies rather than as precise classifications of individual comments. To assess classification consistency, a post hoc manual consistency check was performed on a stratified random sample of 300 comments (100 per category). The agreement between the SnowNLP output and manual coding reached 72.0%, with Cohen’s κ = 0.58, indicating moderate consistency.

Third, the video-text corpus was analyzed to identify persuasive strategies. Chinese text was segmented and filtered by part-of-speech to extract the top 30 most frequent nouns, verbs, and adjectives. These high-frequency lexical items were then manually categorized under Aristotle’s three rhetorical appeals based on both the theoretical meanings of the three appeals and the typical usage contexts observed in KWIC concordance lines. Tokens were not classified solely according to their dictionary meanings, but according to their recurrent rhetorical functions in context. Because the three appeals may overlap in practice, multi-label assignments were allowed when a token recurrently functioned as more than one type of appeal. For frequency-based summaries, token counts were fully included in each assigned category, and proportions were normalized by the summed frequencies across all category assignments.

Coding stability was assessed via a test-retest procedure on the extracted high-frequency lexical items (Top 30 nouns, verbs, and adjectives; N = 90). After an interval, the same token list was re-coded in a randomized order using the same coding logic, without reference to the first-round labels. For reliability calculation, multi-label cases were treated as composite nominal categories (e.g., Ethos+Logos), and N/A was retained as an additional category rather than removed. Agreement between the two rounds was quantified using percent agreement and Cohen’s kappa, indicating substantial stability (percent agreement = 0.88; Cohen’s κ = 0.82; N = 90). To support reproducibility, the processed coding and reliability materials have been deposited in the public Zenodo repository associated with this article.

**Fig 1:**
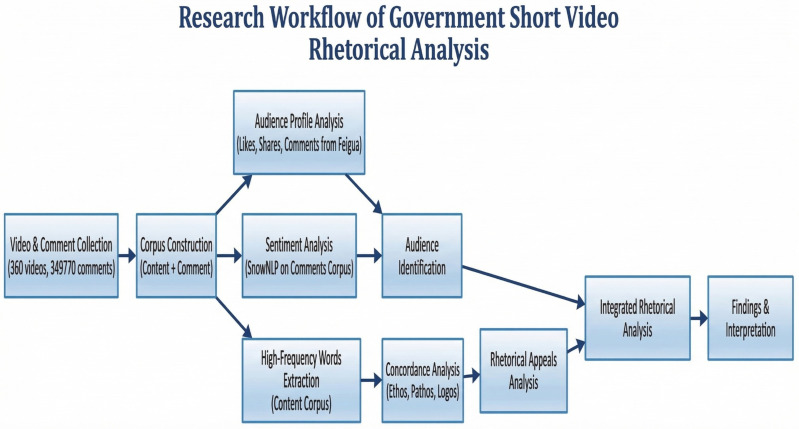
Research workflow integrating corpus-based computation and rhetorical analysis.

Fig 1 summarizes the overall workflow, showing the sequential integration of data collection, computational analysis, and rhetorical interpretation in support of the study’s research objectives.

### 3.4. Ethics statement

This study analyzed publicly available videos and comments from a public Douyin account for academic research. Data were collected from publicly accessible pages without circumventing access controls and, to the best of the author’s knowledge, in compliance with the platform’s terms and conditions. The study involved no interaction with users and collected no direct identifiers (e.g., usernames, user IDs, or profile information). Illustrative examples are presented in de-identified form. Because the study used public data and did not involve identifiable private information, formal ethics approval and informed consent were not required.

## 4. Results and discussion

### 4.1. Audience analysis

Building on the preceding discussion of rhetoric’s audience-centered orientation, this study treats audience information as a core indicator for evaluating the communicative reach and persuasive potential of government short videos. In Burke’s sense of identification [13], persuasion is more likely to succeed when a message resonates with audiences through shared symbols, values, and motives. To characterize the audience profile of *Meili Zhejiang*, three dimensions are examined: basic demographics, expressed attitudes, and interaction patterns. This provides the foundation for examining how rhetorical appeals are realized in the videos (RQ1) and how these appeals resonate with audience dispositions to support engagement and identification (RQ2).

#### (1) Audience demographics.

To ensure temporal alignment between audience demographics and the sampled video content, all statistics reported in this section are based on platform-generated reports retrieved on August 6, 2024, corresponding to the study period. Importantly, while both followers and viewers can serve as indicators of engagement, this analysis draws on viewer data. This choice reflects a rhetorical focus on actual content consumers rather than the broader but more passive base of followers. Viewers more directly represent those exposed to the discourse and thus are more likely to participate in processes of reception and identification, which are central to rhetorical analysis.

According to homepage data retrieved from the official Douyin account “Meili Zhejiang” on August 6, 2024, the account had accumulated 14.575 million followers, published 37,654 videos, and garnered over 874 million likes. These cumulative indicators signal the account’s long-standing visibility and sustained user engagement, positioning it as a prominent case in the government digital communication landscape.

**Fig 2:**
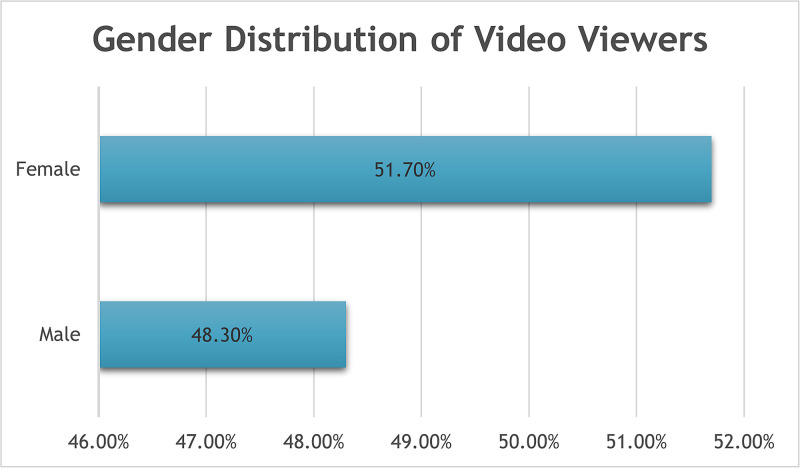
Gender distribution of *Meili Zhejiang* video viewers.

The platform-provided audience statistics reported here serve as contextual indicators of the account’s overall profile rather than individual-level evidence of motivations. Within this scope, demographic patterns offer a useful rhetorical context for understanding how persuasive strategies may be aligned with the audience reached. Regarding gender, viewer distribution exhibits notable parity, with 51.7% female and 48.3% male (Fig 2). This balance suggests that the account’s rhetorical strategies are not tailored to one gender but instead designed to appeal across gender lines. Such a balance creates room for content that integrates ethos, logos, and pathos in ways that are grounded in shared civic and social experience.

Data source: Feigua Data. Notes: Values represent platform-provided estimates for the account during the study period.

**Fig 3:**
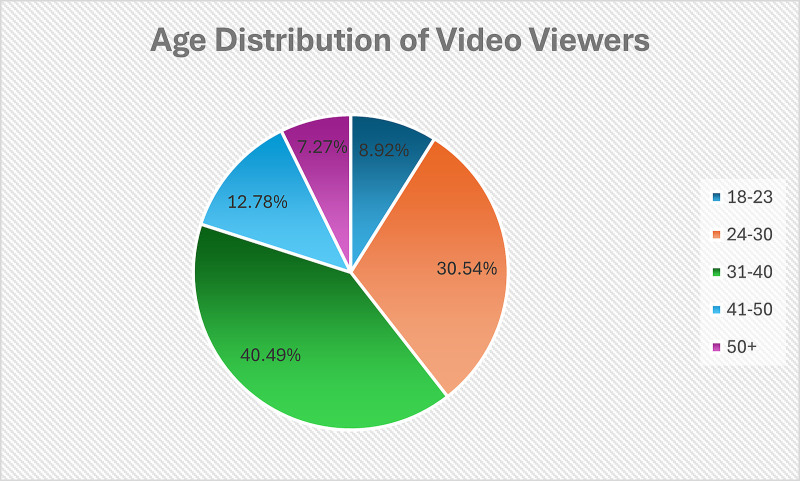
Age distribution of viewers.

By age, the audience is concentrated in the 31–40 age group (40.49%), followed by 24–30 (30.54%) and 41–50 (12.78%) (see Fig 3). Collectively, these three cohorts account for more than 83% of total viewership, indicating that the account primarily reaches working-age users, a group that forms the core of the platform audience in this dataset.

Within this audience context, ethos tends to be particularly persuasive, as working-age audiences often evaluate information through cues of competence, trustworthiness, and responsibility, especially when content concerns public order, safety, or civic services. In this sense, ethos can be understood as a persuasive resource that helps reduce uncertainty and supports identification by presenting institutions, social roles, and public voices as reliable and socially accountable [42,45]. Logos may further support persuasion by providing clear explanations, credible information, and a coherent narrative structure. Yet in the short video context, where audiences consume content rapidly in a condensed form, the space for extended logical elaboration is limited; thus, logos is most effective when information is highly concise and directly relevant [51]. Pathos also plays a prominent role in platform settings, where emotional cues can capture attention and invite interaction under limited time and attention conditions [52,53]. In this study, affective narratives related to local identity, everyday social roles, and community solidarity are treated as plausible routes to identification, consistent with the account’s working-age audience profile.

**Fig 4:**
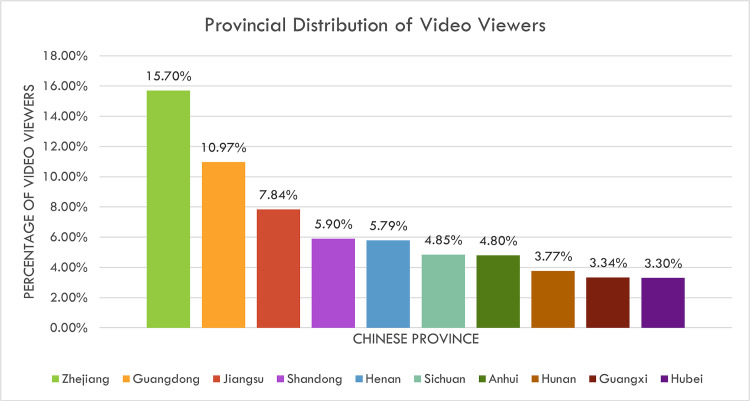
Regional distribution of viewers.

The geographic distribution of video viewers reinforces the account’s strong regional orientation, with Zhejiang province contributing the largest share (15.70%), as shown in Fig 4. Significant viewership also comes from Guangdong (10.97%) and Jiangsu (7.84%), both economically developed coastal regions with high digital engagement and close developmental ties to Zhejiang. Shandong (5.90%) and Henan (5.79%), as two of China’s most populous provinces, likely contribute high viewership due to their large resident populations. Meanwhile, Anhui (4.80%) and Sichuan (4.85%) are major labor-exporting provinces with substantial migrant populations living or working in Zhejiang; their elevated viewer shares may reflect this migratory linkage and enduring regional affinity.

Overall, the gender, age, and regional characteristics of *Meili Zhejiang*’s audience suggest a rhetorically responsive environment. In such a context, ethos, pathos, and logos are selectively deployed to engage a viewership characterized by gender balance, a concentration in working-age groups, and residence in economically and socially integrated regions. These insights provide an empirical foundation for subsequent analyses of how rhetorical appeals function in practice and how they contribute to audience identification.

#### (2) Audience attitudes.

**Fig 5:**
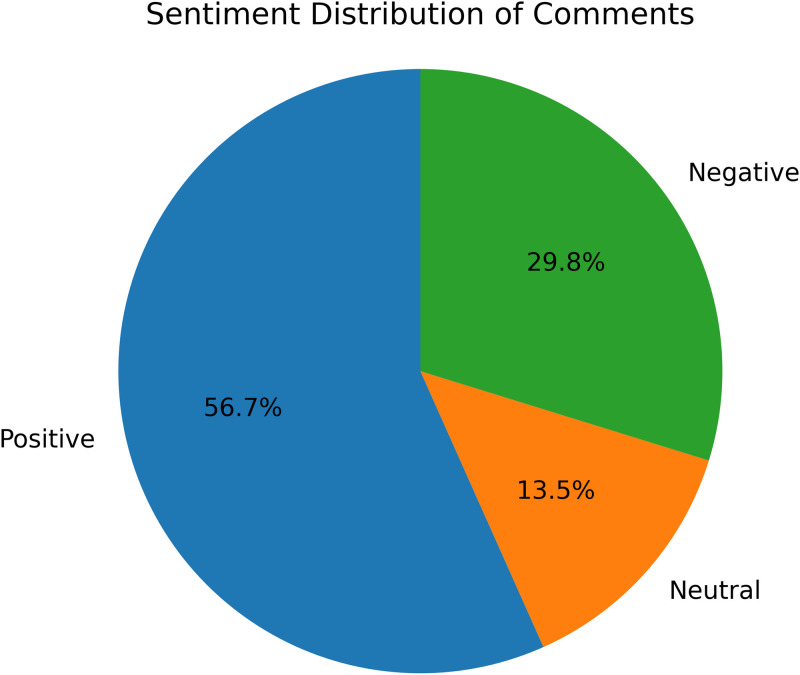
Sentiment analysis of viewer comments.

To assess audience sentiment in the comment corpus surrounding the sampled *Meili Zhejiang* Douyin videos, this study applied the SnowNLP library in Python following the procedure described in the Methodology section. Sentiment scores ranged from 0 to 1: comments scoring above 0.6 were classified as positive, those between 0.4 and 0.6 as neutral, and those below 0.4 as negative. As shown in Fig 5, comments were predominantly positive (56.7%), followed by negative (29.8%) and neutral (13.5%) responses. This distribution suggests a broadly favorable tendency in the observable comment space, while the sizeable negative share indicates that some viewers express concerns or dissatisfaction.

At the same time, a manual consistency check on a stratified sample of 300 comments showed moderate agreement between the SnowNLP output and human coding (72.0% agreement; Cohen’s κ = 0.58). This pattern suggests that, while machine-based sentiment analysis may have difficulty capturing some implicit and context-dependent evaluations, the results remain useful as descriptive indicators of overall audience-attitude tendencies within the observable comment section rather than as precise measurements of individual psychological states.

**Fig 6:**
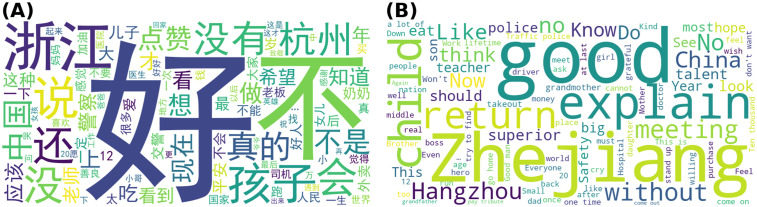
Word clouds of high-frequency terms in the user-comment corpus. **(A) Original Chinese. (B) English translations.** Notes: Word clouds were generated from the study corpus. Translations are provided for readability and do not affect the underlying frequency counts.

Following the procedure described in the Methodology, high-frequency terms in the comment corpus were extracted and summarized with Chinese word clouds and English translations for readability (Fig 6). Positive and supportive reactions are dominated by “grateful” (感谢) and highly affective responses such as “burst into tears” (泪奔), indicating that many viewers frame their responses through appreciation and immediate emotional engagement. Other frequently occurring positive terms reinforce this orientation, including “safety” (平安), “good guy” (好人), “hope” (希望), “like” (喜欢), and “kind” (善良). Together, these lexical cues suggest a discourse of praise, moral affirmation, and optimistic appraisal in which viewers endorse perceived prosocial behavior and desirable outcomes.

Neutral terms mainly refer to identity and contextual descriptors rather than evaluative expressions. High-frequency examples include occupational roles (e.g., “police” (警察), “teacher” (老师), “doctor” (医生)), family roles (e.g., “mother” (妈妈), “grandmother” (奶奶), “son/child” (儿子/孩子)), and locality markers (e.g., “Zhejiang” (浙江), “Hangzhou” (杭州)). Although not inherently affective, these terms indicate the civic, familial, and community domains through which audience identification is commonly articulated.

Negative comments often contain negation or constraint expressions, including “no/not” (没有), “not” (不是), “don’t” (不要), “cannot” (不能), as well as dissatisfaction markers such as “don’t want” (不想) and “not good” (不好). In this corpus, such wording most often functions as an evaluation of the incidents depicted in the videos, the way they are explained or framed, and perceived gaps in information or justification, rather than a rejection of the account as a whole. These critical voices highlight points where clearer context, more concrete detail, or more explicit reasoning could strengthen credibility and better address diverse audience concerns.

#### (3) Audience engagement.

Since the video sample is defined by monthly top-liked posts, audience engagement is already embedded in the selection criterion. For audience-response analysis, all publicly visible comments under each sampled video were collected, and exact-text duplicate comments were removed before analysis. This approach reduces bias introduced by comment ranking and better reflects the observable distribution of audience reactions associated with high-engagement videos.

Likes, comments, and shares not only indicate the reach of Douyin short videos but also reflect the degree of user interaction, serving as rhetorical feedback on the resonance of communicative strategies [54]. During the one-year sampling period from April 1, 2023, to March 31, 2024, the *Meili Zhejiang* account had published approximately 12,000 videos, accumulating 219 million likes, 14.24 million comments, 22.92 million shares, and 10.36 million saves. Compared with liking, commenting typically requires greater cognitive effort and carries higher visibility, which may explain why comment volumes remain significantly lower than like counts. Shares and saves represent additional forms of engagement, indicating interpersonal dissemination and perceived utility for later reference.

These interaction patterns provide context for the rhetorical analysis that follows. High like counts indicate that the sampled videos received relatively high visible audience approval within the platform’s engagement environment, while lower comment volumes suggest that discursive participation is more selective. Nonetheless, the presence of user-initiated feedback through commenting, sharing, and saving suggests that rhetorical appeals operate in an environment of active, though uneven, audience participation. This context underscores the importance of ethos, pathos, and logos in shaping trust, emotional resonance, and shared meaning within the *Meili Zhejiang* digital community.

### 4.2. The three rhetorical appeals in *Meili Zhejiang* videos

**Fig 7:**
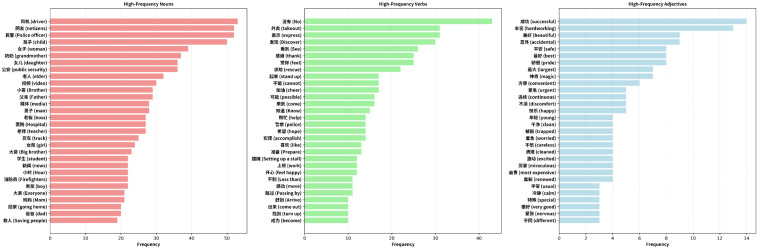
Top 30 nouns, verbs, and adjectives in the video-text corpus (Chinese tokens with English translations).

This section analyzes how the short videos from *Meili Zhejiang* mobilize the three Aristotelian rhetorical appeals, namely ethos, pathos, and logos, to engage their audience. A corpus-assisted lexical analysis was conducted by extracting the top 30 high-frequency nouns, verbs, and adjectives from the corpus and categorizing them by their dominant rhetorical function. In line with the theoretical definitions discussed above, lexical items were coded according to whether they primarily contributed to credibility construction, emotional appeal, or factual and logical grounding. Specific subcategories were further identified from repeated contextual patterns in the concordance lines and are reported in Table 1. Because the three appeals often co-occur in practice, multi-label assignments were permitted when a token recurrently served more than one appeal in its concordance contexts. Fig 7 presents the top 30 nouns, verbs, and adjectives in the original Chinese with English translations, and Table 1 reports their rhetorical categorization.

**Table 1:** Categorization of high-frequency words according to ethos, logos, and pathos. Note: Frequency values indicate occurrences in the corpus. Words are categorized according to their dominant rhetorical function.

Appeal	Subcategory	Sample nouns (freq.)	Sample verbs (freq.)	Sample adjectives (freq.)	Total frequency	Proportion
**Ethos**	Authority	police officer (52), public security (36), teacher (27), media (28), hospital (27)	express (31)		201	15.22%
	Credibility	netizens (52), boss (27) driver (53),	accomplish (14), prepare (13)	successful (14)	173	13.11%
	**Subtotal (Ethos)**				**374**	**28.33%**

**Pathos**	Love and Warmth	child (50), daughter (36), mom (21), dad (20), elder (32), big brother (23), going home (20)	like (13), move (11), feel happy (12)	beautiful (9), young (4), happy (5)	256	19.39%
	Encouragement and Support	everyone (21), firefighters (22), saving people (19)	encourage (25), thank (25), help (14), arrive (10)	safe (8), convenient (6) hardworking (13)	163	12.35%
	Pride and Achievement	boy (22), girl (24), student (22)	move (11), hope (14)	proud (8), excited (4), successful (14), miraculous (4), very good (3), best (8)	134	10.15%
	Fear and Urgency		rescue (22)	urgent (5), trapped (4), nervous (3), careless (4)	38	2.88%
	**Subtotal (Pathos)**				**591**	**44.77%**

**Logos**	Factual Information	netizens (52), driver (53), news (22), video (30), man (28), woman (39)	discover (30), express (31), become (10)		295	22.35%
	Precision	hour (22)	know (15), less than (11)	largest (7), continuous (5)	60	4.54%
	**Subtotal (Logos)**				**355**	**26.89%**
	**Total**				**1320**	**100%**

#### 4.2.1. Ethos: authority and credibility.

*Meili Zhejiang* constructs ethos primarily through recurring credibility cues in videos that receive high levels of audience endorsement. Feigua Data (see Fig 3) shows that the account’s viewers are largely concentrated among adults aged 24–40, which provides essential contextual background for interpreting audience alignment in this platform setting. Within the corpus, institutional trustworthiness, ethical conduct, and civic responsibility emerge as salient rhetorical touch points through which ethos is articulated. Accordingly, the account’s ethos is reinforced through three interrelated strategies: asserting institutional authority, incorporating relatable civilian perspectives, and highlighting ethical actions with positive social consequences.

**Fig 8:**
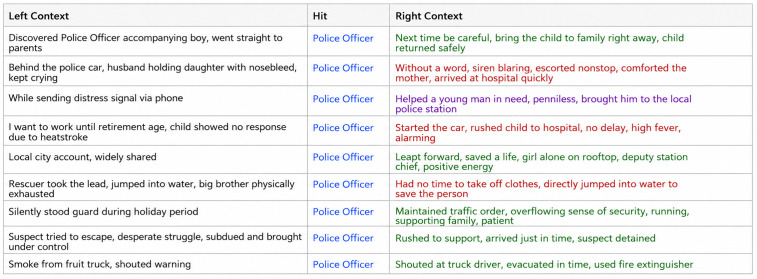
Concordance lines for “police officer” (translated from the Chinese corpus). Notes: Figs 8-16 present KWIC-style concordance lines exported from AntConc based on the Chinese corpus. In each row, the “Hit” column shows the keyword, while “Left Context” and “Right Context” display the surrounding words within the context window configured in AntConc. English translations are provided for readability and do not affect the quantitative analyses.

First, as an official communication outlet of the Zhejiang provincial government, *Meili Zhejiang* enjoys inherited institutional authority. This foundational ethos is reinforced through frequent references to trusted entities such as police officers, public security bureaus, and teachers, roles that align with the audience’s civic awareness and high valuation of public service. Concordance analysis of the term “police officer” (see Fig 8) illustrates frequent co-occurrence with service-oriented expressions such as “saved a life,” “evacuated in time,” and “rush to support.” These portrayals depict law enforcement not merely as enforcers of authority, but as compassionate and protective figures embedded in everyday social life. Such portrayals are consistent with a strategic alignment between credibility cues and affective framing, which may facilitate trust and identification in a short-video platform setting.

**Fig 9:**
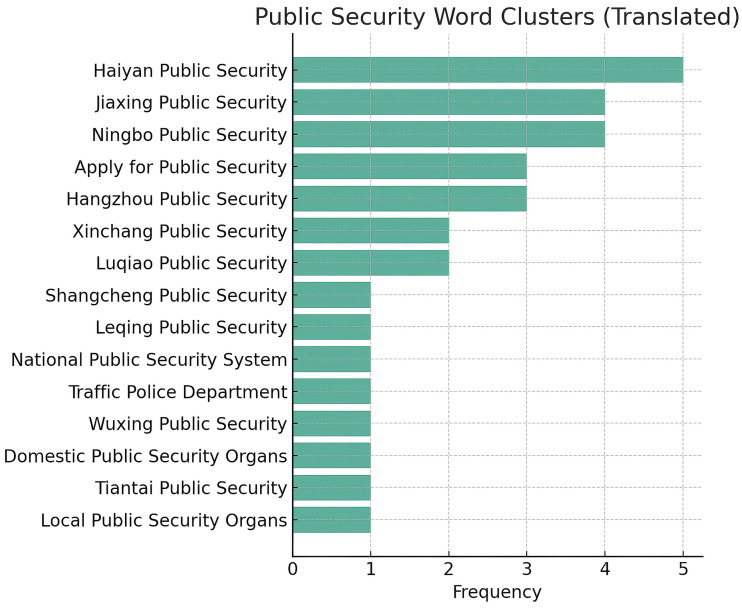
Lexical cluster of “public security” with regional identifiers (translated from the Chinese corpus).

This alignment between institutional credibility and local relevance is further illustrated through lexical clustering analysis. As shown in Fig 9, the term “public security” frequently co-occurs with specific geographic identifiers such as “Haiyan,” “Jiaxing,” “Ningbo,” and “Hangzhou.” These references indicate that the sources of the videos are often tied to local official media outlets and local official departments [50]. They also reveal a broad spatial distribution of content across multiple regions within Zhejiang province. This pattern aligns closely with the geographic profile of the platform’s primary audience, the majority of whom are concentrated in Zhejiang itself. By embedding institutional discourse within familiar local contexts, these videos reinforce both the credibility and relevance of their messaging, fostering a stronger sense of identification among regional viewers.

**Fig 10:**
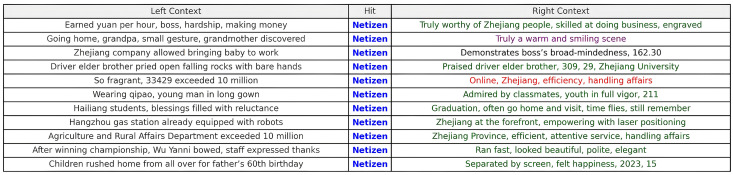
Concordance lines of “netizen” (translated from the Chinese corpus).

In addition to asserting official authority, the account reinforces its credibility by incorporating the perspectives of ordinary citizens. This participatory dimension is also reflected in the videos’ sourcing, which includes contributions from netizens and members of the public [50]. Terms such as “netizen” and “boss” function as discursive proxies for public voices or eyewitness accounts, lending a sense of authenticity and objectivity to the overall narrative. The concordance of “netizen” (see Fig 10) illustrates how such voices appear in ethically framed contexts, such as “truly a warm and smiling scene,” “boss’s broad-mindedness,” and “Zhejiang at the forefront.” By framing institutional events through the lens of public commentary, these videos enhance the relatability and trustworthiness of their content. This rhetorical move reframes state narratives not merely as top-down communication but also as grounded in lived experience, thereby forging a hybrid ethos that blends official authority with socially validated credibility.

Taken together, these rhetorical strategies demonstrate that *Meili Zhejiang* constructs ethos not as a static projection of authority, but as a dynamic interplay of credibility, locality, and ethical legitimacy, carefully aligned with the social positions, moral expectations, and media habits of its target audience.

As noted in the previous section, ethos can sometimes be reinforced through discrediting moves that cast doubt on alternative actors or interpretations. However, such discrediting-oriented cues are not salient in the present corpus for three reasons. First, *Meili Zhejiang* is designed to disseminate positive and socially affirmative content and promote a favorable image of Zhejiang, which encourages an exemplary and harmonious tone [50]. Second, the dataset is drawn from monthly top-liked videos, which prioritize audience-endorsed content and therefore tend to amplify positive ethos cues. Third, because the account addresses a broad national audience, overt discrediting of other institutions or accounts is unlikely to function as an effective persuasive strategy in this setting. Given the study’s lexical focus, any subtle conflict, if present, may also be expressed through implied contrasts, attribution patterns, or other discursive features rather than overtly negative wording. This boundary clarifies the scope of the current findings and suggests avenues for future research.

#### 4.2.2. Pathos: emotional resonance and identification.

According to lexical frequency analysis, pathos is the most prominent rhetorical strategy, accounting for 44.77% of the emotionally significant expressions across the selected videos. It operates through four key subcategories: Love and Warmth, Encouragement and Support, Pride and Achievement, and Fear and Urgency, each serving a distinct affective function in supporting audience engagement and identification.

(1) Love and warmth

**Fig 11:**
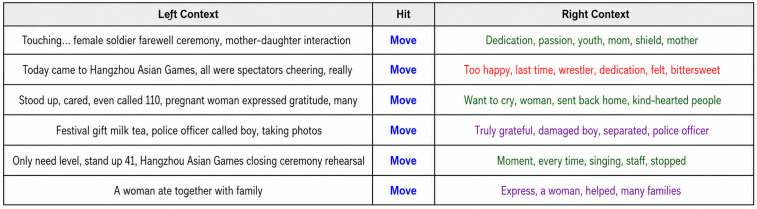
Concordance lines of “move” (translated from the Chinese corpus).

**Fig 12:**
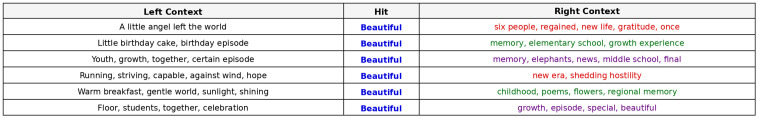
Concordance lines of “beautiful” (translated from the Chinese corpus).

This subcategory features frequent use of terms such as “child,” “daughter,” “mom,” “dad,” and “elder,” evoking close familial bonds and affection. Verbs such as “like” and “move,” and adjectives such as “young,” “beautiful,” and “happy” contribute to a warm, emotionally resonant atmosphere. As shown in the concordance of “move” (Fig 11), these expressions are embedded in contexts of compassion, gratitude, and communal care. Similarly, the concordance of “beautiful” (Fig 12) reveals associations with youth, hope, and cherished memories, as in “childhood poems and flowers,” “new era,” and “growth.” These word clusters foreground not only familial intimacy but also a broader sense of generational continuity and collective well-being. In doing so, they reinforce a narrative of social harmony and mutual care, appealing to universal human experiences and encouraging viewers to identify personally and emotionally with the presented narratives.

(2) Encouragement and support

**Fig 13:**
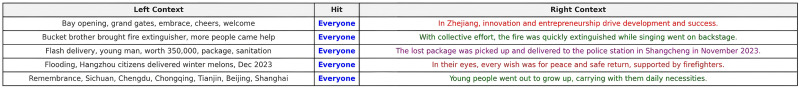
Concordance lines of “everyone” (translated from the Chinese corpus).

This category is manifested through the frequent portrayal of community-oriented roles such as “everyone” (the general public) and “firefighters.” These figures symbolize the collective strength of society and the willingness of individuals from diverse backgrounds to contribute to the common good. Verbs like “encourage,” “thank,” and “help” highlight the importance of mutual assistance and collaboration, fostering a discourse of unity and shared responsibility. Adjectives such as “safe” and “convenient” further underscore the value of these contributions, positioning the community as both resilient and compassionate. As illustrated in the concordance of “everyone” (Fig 13), the term frequently appears in contexts of collective action and moral responsibility, for instance, “quickly extinguish fire,” “deliver a lost package to a police station,” or “supported by firefighters.” These associations construct the community as a reliable force for good, underscoring solidarity and civic participation. By emphasizing such qualities, the videos cultivate a sense of cohesion and shared pride, inviting viewers to perceive themselves not only as passive observers of public life in Zhejiang but as active, valued participants in the collective life of their communities.

(3) Pride and achievement

**Fig 14:**
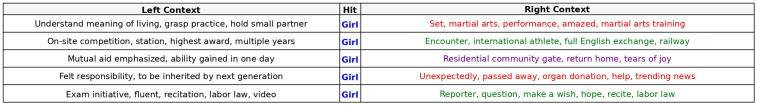
Concordance lines of “girl” (translated from the Chinese corpus).

This category emphasizes both personal and collective accomplishments. Terms such as “boy” and “girl” not only designate individuals who achieve success but also symbolize the future promise and potential of Zhejiang’s younger generation. Verbs like “hope” and “move” convey aspiration, perseverance, and the pursuit of dreams, while adjectives including “proud,” “excited,” and “successful” celebrate concrete achievements. Expressions such as “miraculous” and “very good” further underscore the extraordinary nature of these accomplishments. As illustrated in the concordance of “girl” (see Fig 14), the term appears in contexts that foreground youth achievement and aspiration, such as “martial arts training,” “full English exchange,” and “make a wish.” These associations highlight both individual determination and cultural pride, framing young women as an embodiment of vitality, hope, and collective progress.

(4) Fear and urgency

This category captures the pressing and serious dimensions of community life, particularly emergency situations. Terms such as “rescue” and “urgent” underscore the immediacy of action required in critical moments, while adjectives like “trapped” and “nervous” evoke the tension and vulnerability inherent in such contexts. By representing these scenarios, the videos raise awareness of safety and preparedness while simultaneously eliciting empathy and concern from viewers. At the same time, depictions of swift and effective responses by multiple actors serve to mitigate anxiety, reinforcing a sense of security and trust in collective crisis management.

#### 4.2.3. Logos: factuality and logical reasoning.

Logos in the selected short videos operates through a consistent emphasis on factuality and precision. First, the frequent appearance of terms such as “driver,” “news,” and “man” reflects a grounding of narratives in the perspectives of eyewitnesses and everyday individuals. Anchoring the discourse in concrete and observable realities enhances the perceived objectivity of the message. The inclusion of social referents, such as the term “netizens,” further situates these accounts within a recognizable public sphere, which increases their relatability. Verbs of observation, reporting, and response, such as “discover” and “express,” further present the content as rooted in real-world experience rather than abstract assertion.

**Fig 15:**
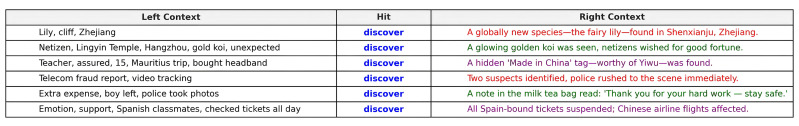
Concordance lines of “discover” (translated from the Chinese corpus).

The concordance lines for the verb “discover” (Fig 15) illustrate this pattern. It appears in contexts involving rare species, unexpected sights, or personal realizations. Phrases such as “discovered fairy lilies on a cliff” or “discovered glowing koi fish at Lingyin Temple” position the speaker as a direct witness to reality. This framing reinforces the factual grounding of the narrative and highlights the reliability of what is seen and shared. By drawing attention to immediate and clearly observable events, such expressions show how logos operates in these short videos: they transform individual experiences into collective knowledge claims, thereby lending the discourse a sense of precision and credibility.

**Fig 16:**
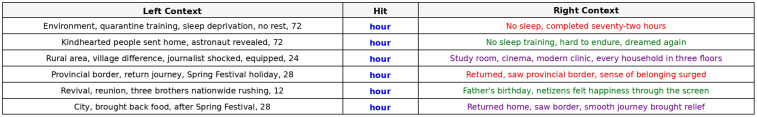
Concordance lines of “hour” (translated from the Chinese corpus).

Second, the use of quantifiable or specific vocabulary, such as “hour,” “largest,” and “continuous,” strengthens the precision of the discourse. These lexical choices enhance the specificity of the videos’ narratives, promoting clarity and informational accuracy. For example, the concordance of the noun “hour” (Fig 16) frequently pairs with exact durations. These instances, such as “72 hours of sleep deprivation training” or “28-hour return trip during Spring Festival,” lend temporal specificity to the narratives. By integrating such verifiable data points and situating experiences in measurable terms, the videos build a rational framework that appeals to the audience’s sense of logic, complementing the emotional and ethical appeals outlined in previous sections.

### 4.3. Integrative analysis: aligning three appeals with audience identification

The analysis of top-performing videos from *Meili Zhejiang* indicates a patterned construction of emotional, ethical, and logical appeals that aligns with the account’s audience profile and platform environment. Corpus-based evidence suggests that pathos, ethos, and logos work together to support affective resonance, institutional trust, and informational plausibility, thereby strengthening audience identification in government short-video communication.

Pathos remains the most salient appeal, accounting for 44.77% of the high-frequency lexical items categorized as emotionally oriented. Emotionally charged nouns, verbs, and adjectives cluster around four affective domains: familial warmth, encouragement, collective pride, and urgency. This affect-rich framing is consistent with the account’s core audience profile, especially viewers aged 24–40 (Fig 3), and with the selected videos’ emphasis on relatable, human-interest storytelling. The predominance of positive sentiment in comments (56.7%) is also consistent with the pathos-centered content profile identified in the corpus, suggesting that in the context of government short videos, affective appeals are associated with audience emotional resonance and favorable response patterns observed in the data.

Ethos (28.33%) and logos (26.89%) appear at similar levels in the distribution and together provide key support for persuasion alongside pathos. These expressions often appear in service-oriented and ethically resonant contexts, linking authority to everyday civic life in ways that are broadly consistent with the civic concerns of viewers in Zhejiang and neighboring provinces. At the same time, a hybrid ethos that combines official authority with grassroots validation bolsters both the credibility and resonance of the message. This pattern is echoed in the neutral identity terms found in viewer comments, especially occupational and family roles. By embedding authority within these socially recognized identities, *Meili Zhejiang* develops a dual-layered credibility strategy that anchors its discourse in both institutional legitimacy and everyday social experience, thereby supporting identification with a regionally rooted, socially engaged audience.

Logos functions as an informational and evidential anchor. It is realized through lexemes associated with factual observation and quantifiable precision. These linguistic features enhance narrative realism and provide cognitive anchors for emotional and ethical appeals. For a short-video audience navigating rapid, platform-mediated consumption, this synthesis of evidence and emotion may bolster perceived plausibility and facilitate persuasion. At the same time, sentiment analysis shows a substantial minority of critical comments raising concerns or dissatisfaction, suggesting that logos-related support could be strengthened. Providing clearer context and more explicit factual grounding may help sustain credibility and reduce the risk that persuasive messaging is interpreted as relying mainly on emotional appeal.

Overall, the results suggest a patterned construction in which pathos drives emotional resonance, while ethos and logos provide parallel support through credibility cues and informational grounding.

This pattern can be understood through the interaction between rhetorical theory and the communicative logic of short-video platforms. From the perspective of Burkean identification, audiences are more likely to align with institutional discourse when public issues are translated into shared values and emotions, familiar social roles, and recognizable everyday situations. Rather than presenting governance as an abstract administrative process, *Meili Zhejiang* frequently embeds institutional messages in scenes of ordinary people, local places, emergency assistance, and moral action. Such textual choices reduce symbolic distance between the government account and its audience, thereby making identification more attainable. At the same time, Aristotle’s three appeals clarify why persuasion depends on their coordinated operation rather than on any single appeal alone: emotional salience attracts attention and invites immediate response, while ethos and logos stabilize persuasive meaning by providing institutional credibility and factual plausibility. This interplay reflects strategic audience alignment under the sociotechnical conditions of short-video platforms, where limited attention and engagement-based visibility favor concise, emotionally legible narratives that remain credible and factually anchored. However, the prominence of emotional appeal also points to a potential limitation. Over-reliance on pathos may narrow the informational and rational depth of public discourse, shifting attention toward affective immediacy at the expense of clarity and credibility. In government communication, where transparency and trust are essential, this suggests the need for a more calibrated integration of emotional resonance, factual grounding, and institutional voice.

## 5. Conclusion

This study examined how government short videos mobilize rhetorical resources by analyzing a year-long sample of *Meili Zhejiang*’s 360 most-liked videos and all publicly visible comments associated with them. Using corpus-assisted lexical analysis, Python-based frequency and sentiment analysis, and rhetorical interpretation, this study mapped the distribution of Aristotle’s three appeals and examined their alignment with audience profiles and engagement patterns. The analysis addressed how ethos, pathos, and logos are realized within the discursive dimension of government short-video discourse and how their constructions align with observable attention and participation patterns that support audience identification.

Empirically, the findings show that pathos predominates (44.77%) through affect-rich nouns, verbs, and adjectives that evoke familial warmth, community spirit, pride, and urgency. This emotional framing is strategically aligned with short-video platform dynamics, where salient human-interest cues can invite immediate resonance and participation. Ethos (28.33%) is shaped through institutional roles and local references that ground authority in civic life while incorporating citizen voices for authenticity. Logos (26.89%) remains closely aligned with ethos in overall prominence and functions as an informational anchor by supplying concise factual expressions and quantifiable details that stabilize interpretation and support credibility. Together, these patterns point to strategic audience alignment in a short-video setting: emotionally salient storytelling draws viewers in, ethos-based credibility cues foster trust, and logos-based factual grounding sustains plausibility under rapid, platform-mediated consumption. Feigua Data (Fig 3) indicates that the account’s viewers are primarily concentrated among working-age adults. The predominance of positive sentiment in comments (56.7%) is broadly consistent with the rhetorical configuration observed in the videos, suggesting that this combination of emotional salience, credibility cues, and informational clarity is associated with a generally favorable reception in the observable discourse.

Theoretically, the study highlights government short videos as inherently rhetorical acts whose effectiveness lies in the dynamic integration of emotional, ethical, and logical appeals. Their persuasive force derives less from isolated strategies than from the calibrated interplay between discourse design and audience dispositions. Methodologically, this research demonstrates how corpus-based investigation combined with rhetorical analysis can uncover persuasive patterns in the textual dimension of digital governance communication. Practically, the findings suggest that producers may benefit from sustaining this synergy by balancing emotional resonance with institutional authority and factual clarity, leveraging pathos to attract, ethos to stabilize, and logos to consolidate message legitimacy.

Nonetheless, this study is limited by its focus on a single provincial-level government account on one platform, the monthly top-liked sampling design, and a lexical analysis conducted within a Chinese cultural context. Because the sample is engagement-based, the findings characterize rhetorical patterns in high-approval and highly visible content rather than the account’s full routine output. Although all publicly visible comments under each sampled video were included, the observable discourse is inherently subject to platform-specific participation norms and moderation practices. These constraints may affect the generalizability of the findings. Future research could extend the design through cross-platform and cross-regional comparisons, broader content sampling, longitudinal analysis, and, where appropriate, systematic multimodal approaches.
